# A 2.8 kV Breakdown Voltage α-Ga_2_O_3_ MOSFET with Hybrid Schottky Drain Contact

**DOI:** 10.3390/mi15010133

**Published:** 2024-01-14

**Authors:** Seung Yoon Oh, Yeong Je Jeong, Inho Kang, Ji-Hyeon Park, Min Jae Yeom, Dae-Woo Jeon, Geonwook Yoo

**Affiliations:** 1 Department of Intelligent Semiconductor, Soongsil University, Seoul 06938, Republic of Korea; osy1549@gmail.com; 2 School of Electronic Engineering, Soongsil University, Seoul 06938, Republic of Korea; wjddudwpp@gmail.com (Y.J.J.); duaalswo94@gmail.com (M.J.Y.); 3 Power Semiconductor Research Division, Korea Electrotechnology Research Institute, Changwon 51543, Republic of Korea; ihkang@keri.re.kr; 4 Korea Institute of Ceramic Engineering & Technology, Jinju 52851, Republic of Korea; jhp5511@kicet.re.kr (J.-H.P.); dwjeon@kicet.re.kr (D.-W.J.)

**Keywords:** α-Ga_2_O_3_, MOSFET, hybrid Schottky drain, Ohmic drain, breakdown voltage

## Abstract

Among various polymorphic phases of gallium oxide (Ga_2_O_3_), α-phase Ga_2_O_3_ has clear advantages such as its heteroepitaxial growth as well as wide bandgap, which is promising for use in power devices. In this work, we demonstrate α-Ga_2_O_3_ MOSFETs with hybrid Schottky drain (HSD) contact, comprising both Ohmic and Schottky electrode regions. In comparison with conventional Ohmic drain (OD) contact, a lower on-resistance (R_on_) of 2.1 kΩ mm is achieved for variable channel lengths. Physics-based TCAD simulation is performed to validate the turn-on characteristics of the Schottky electrode region and the improved R_on_. Electric-field analysis in the off-state is conducted for both the OD and HSD devices. Furthermore, a record breakdown voltage (BV) of 2.8 kV is achieved, which is superior to the 1.7 kV of the compared OD device. Our results show that the proposed HSD contact with a further optimized design can be a promising drain electrode scheme for α-Ga_2_O_3_ power MOSFETs.

## 1. Introduction

Gallium oxide (Ga_2_O_3_) semiconductors have been rigorously studied for use in high-performance power devices owing to their ultra-wide bandgap (E_g_) and to the high-quality Ga_2_O_3_ substrates obtained from a bulk single crystal using melt-growth methods [1,2,3,4]. Interestingly, Ga_2_O_3_ crystalizes into five polymorphic phases, and the thermodynamically stable β-Ga_2_O_3_ has stood at the center of research. On the other hand, the second most well-known α-Ga_2_O_3_ has certain advantages because of its epitaxial compatibility with commercially available, large-size sapphire wafers. Furthermore, the heteroepitaxy of high-quality α-Ga_2_O_3_ epilayers on sapphire will allow better thermal conductivity compared with a native substrate [5,6,7,8,9]. It is to be noted that α-Ga_2_O_3_ has the highest E_g_ of ~5.3 eV among Ga_2_O_3_ phases and a correspondingly competitive figure of merits. Thus, α-Ga_2_O_3_ deserves to be extensively investigated further.

Attributed to its high E_g_, α-Ga_2_O_3_ has received great interest for ultraviolet (UV) light detectors and specifically deep-UV or UV C-band light (<280 nm) [10,11]. Among various materials with a wide E_g_, including aluminum gallium nitride (AlGaN) and zinc oxide (ZnO), α-Ga_2_O_3_ is particularly appropriate for large-area processes because of its relatively low growth temperature of about 500 °C. This relatively low temperature of growth or deposition allows not only the use of conventional large-area process methods but also novel Ga_2_O_3_ film deposition approaches via thermal oxidation of Ga-based wafers [12,13,14]. These unique advantages of α-Ga_2_O_3_ along with large-area film growth techniques make α-Ga_2_O_3_ promising for use in high-performance UVC and flame photodetectors.

Power devices based on α-Ga_2_O_3_ represent another important application. Indeed, various α-Ga_2_O_3_-based device structures have been reported. For example, Schottky barrier diodes using mist-CVD-grown α-Ga_2_O_3_ films and α-Ir_2_O_3_/α-Ga_2_O_3_ hetero p-n junction diodes have been demonstrated [15,16]. Metal-semiconductor field-effect transistors based on a mist-CVD-grown α-Ga_2_O_3_ layer on sapphire were also proposed [17]. Jeong et al. reported an α-Ga_2_O_3_ MOSFET with a breakdown voltage (BV) of 2.3 kV grown via halide vapor-phase epitaxy (HVPE) [18]. Considering the demonstrated α-Ga_2_O_3_ devices’ structures, further enhancement of on-resistance (R_on_) and breakdown characteristics can be achieved not only through a vertical structure but also through additional fabrication process steps such as surface passivation and field-plate structures [19,20,21,22,23,24]. Alternatively, the hybrid Schottky–Ohmic drain (HSD) scheme utilizing charge trapping in the Schottky extension region can be considered because of the relatively inferior crystal quality of heteroepitaxial α-Ga_2_O_3_ compared to the homoepitaxial Ga_2_O_3_ layer [25,26,27,28]. Indeed, the use of HSD on β-Ga_2_O_3_ FETs has been reported to alleviate degradation under thermal stress conditions [29].

In this work, for the first time, we propose α-Ga_2_O_3_ MOSFETs with HSD electrodes. The heteroepitaxial α-Ga_2_O_3_ device layer was grown on a sapphire substrate via HVPE. The HSD is composed of Ti/Al/Ni/Au Ohmic and Ni Schottky electrodes, and both contact regions are analyzed using high-resolution transmission electron microscopy (HR-TEM). The electrical characteristics of the proposed HSD device are compared with a conventional Ohmic drain (OD) device for variable source–drain lengths (L_SD_). Moreover, physics-based TCAD simulation is performed to elucidate the turn-on characteristics of the proposed HSD device. Electric (E)-field simulation in the off-state is also performed to compare the OD and HSD devices. Finally, the off-state BV of 2.8 kV is measured for the HSD device. The performance is compared with recently reported results.

## 2. Experimental Section

Heteroepitaxial α-Ga_2_O_3_ films were deposited on a 2-inch (0001) sapphire substrate using the HVPE method with GaCl and O_2_ precursors at a growth temperature of 470 °C. Undoped (~900 nm) and Si-doped α-Ga_2_O_3_ (~300 nm) layers were grown for 10 min each, with SiH_4_ gas supplied as an n-type dopant during the growth of the Si-doped layer. Donor concentration, Hall mobility, and sheet carrier density of the Si-doped α-Ga_2_O_3_ epitaxial layer were measured to be 6 × 10^17^ cm^−3^, 98.7 cm^2^/V∙s, and 1.8 × 10^13^ cm^−3^, respectively. Surface cleaning was performed for 10 min on each layer using acetone and IPA. The MESA isolation structure was then patterned using BCl_3_/Ar gas in the appropriate ratio with ICP RIE. The source/drain electrodes were deposited using an e-beam evaporator with a Ti/Al/Ni/Au (25/140/40/100 nm) metal stack and patterned using conventional photolithography and lift-off processes. Rapid thermal annealing was carried out in a N_2_ ambient at 470 °C for 1 min to improve contact properties. The Schottky drain electrode of Ni (100 nm) is deposited by a DC sputtering system for the HSD device fabrication process. A 20 nm thick HfO_2_ gate dielectric layer was deposited as the gate oxide using atomic layer deposition at 350 °C with a deposition rate of 1 Å/cycle using Tetrakis (ethylmethy lamino) hafnium precursor and ozone reactant. Finally, the Ni (100 nm) gate electrode was deposited by sputtering, followed by opening contact holes via ICP RIE.

Scanning electron microscopy images and cross-sectional TEM samples were prepared using a dual-beam focused ion beam (FIB-SEM, Helios 460F1). The TEM images and energy dispersive X-ray spectroscopy (EDS) elemental maps were obtained using a JEOL JEM-2100F with a probe Cs corrector. All electrical measurements were conducted using a semiconductor parameter analyzer (Keithley 4200A-SCS) in a dark box. The off-state breakdown measurement was conducted using in-house test equipment composed of a 5 kV power supply (Tektronix model 2290-5), picoammeter (Tektronix model 6485), source-measurement unit (Tektronix model 2440), and protection circuit. Physics-based TCAD (Synopsys, Inc., Mountain View, CA, USA) simulation was performed by elaborately reflecting parameters of the fabricated devices.

## 3. Results and Discussion

**Figure 1:**
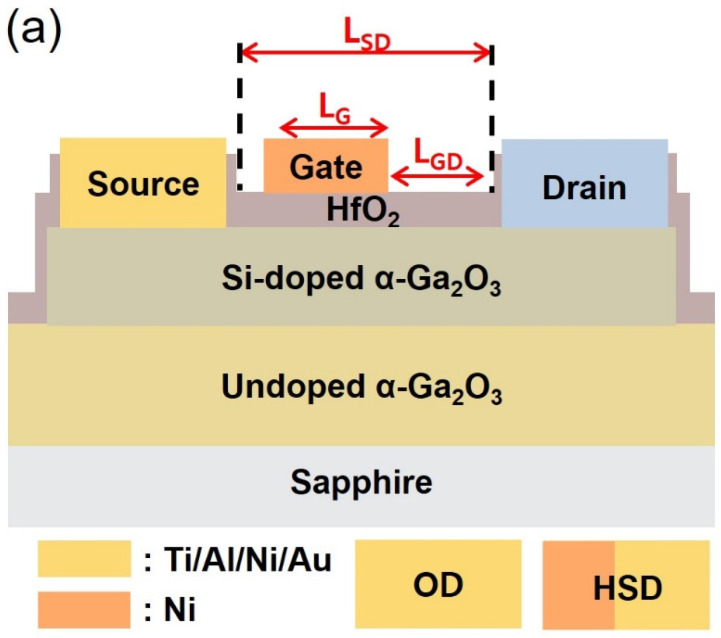

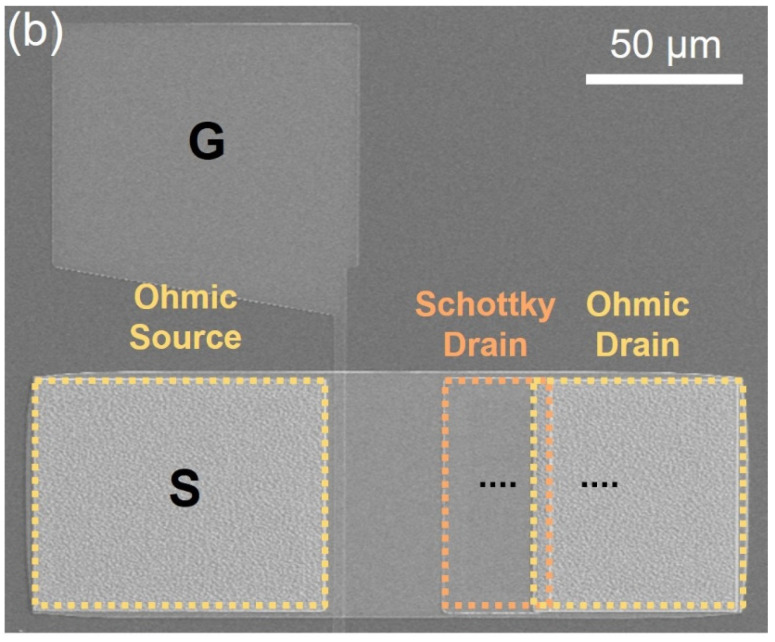
(**a**) A cross-sectional schematic and (**b**) top-view scanning electron microscope image of the fabricated α-Ga_2_O_3_ MOSFET with HSD contact. The blue box represents the drain electrode of OD or HSD. Dashed lines (black) show the region of the focused-ion beam milling for HR-TEM analysis.

**Figure 2:**
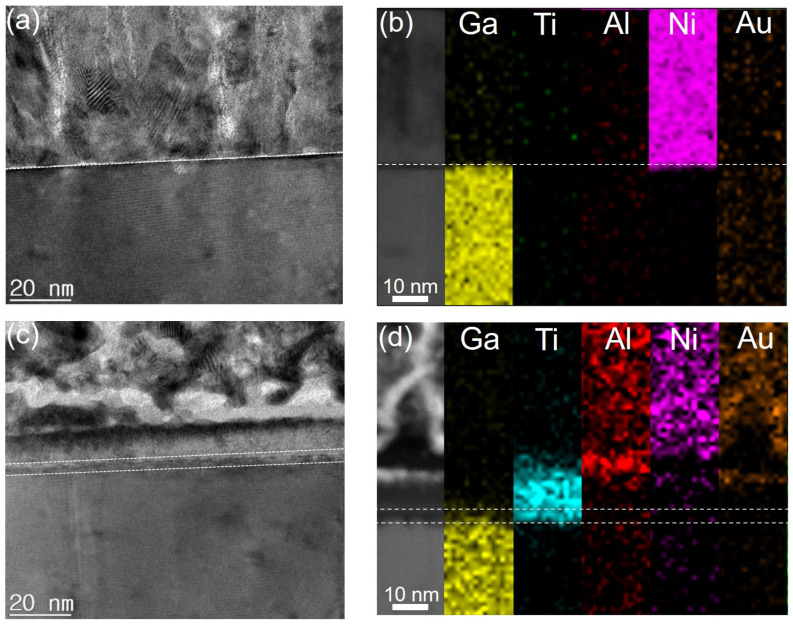
HR-TEM images and corresponding EDS elemental mapping images of the (**a**,**b**) Schottky and (**c**,**d**) Ohmic contact regions.

Figure 1a,b show the cross-sectional schematics and a top view of the representative HSD device, respectively. The blue box represents the drain electrode of the OD or HSD. The fabricated α-Ga_2_O_3_ MOSFETs have a channel-width (W) = 96 μm with a variable L_SD_ of 20, 28, and 40 µm. The gate length (L_G_) and source-gate length (L_SG_) are 2 and 4 µm, respectively. The width of the OD electrode is 96 µm. For the HSD, the Schottky and Ohmic contact regions are designed to be 34 and 62 µm, respectively. The Schottky contact metal is overlaid onto the Ohmic metal by 5 µm. The dashed line shown in Figure 1b indicates the position where TEM images were obtained. Figure 2 compares the TEM and EDS analyses for the Schottky contact (α-Ga_2_O_3_/Ni junction) and Ohmic contact (α-Ga_2_O_3_/Ti/Al/Ni/Au) regions. Based on the reported band alignment analysis, the Schottky contact between Ni and α-Ga_2_O_3_ is predicted. The following equation is used to calculate E_C_-E_F_ and N_C_ [30]:

(1) $$E_{C}-E_{F}=kTln(\frac{N_{C}}{N_{D}})$$

(2) $$N_{C}=2{\left(\frac{2\pi m^{*}kT}{h^{2}}\right)}^{\frac{3}{2}}$$

where N_C_, N_D_, m_0_, and m* are the effective density of states of the conduction band, electron density of α-Ga_2_O_3_, electron mass, and electron effective mass of α-Ga_2_O_3_, respectively. Haiying et al. reported that the value of m* for α-Ga_2_O_3_ is 0.276 m_0_ [31]. The calculated N_C_ is 3.65 × 10^18^ cm^−3^, and the extracted E_C_-E_F_ is 0.05 eV. The work function of Ni (Φ_Ni_) is 5.01 eV, and the electron affinity of α-Ga_2_O_3_ (χ_α-Ga2o3_) is 3.62 eV [32,33]. Therefore, the barrier height formed in Ni/α-Ga_2_O_3_ is calculated to be Φ_Ni_ − χ_α-Ga2o3_ − (E_C_-E_F_) = 1.34 eV. The interface at the Schottky contact shows a smooth and sharp transition. Although we cannot elaborate it quantitively, the ALD temperature does not have a significant impact on the Schottky contact, as observed from electrical characteristics at a low drain bias. It is reported that interdiffusion is marginal under an annealing temperature of 400 °C and that the Ni content was only 5% in the Ni-Au alloy [34]. The Ohmic contact exhibits a relatively mixed interface due to the formation of a Ti-Al inter-metallic phase, and oxygen vacancies facilitated the Ohmic contact by reducing contact resistance [35].

**Figure 3:**
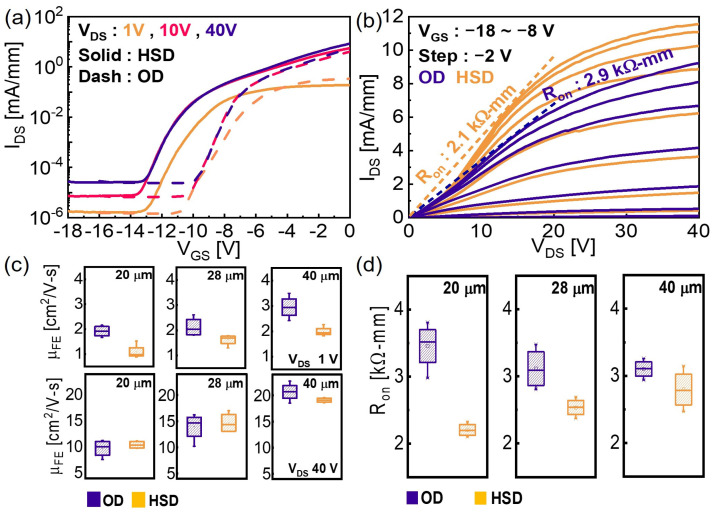
(**a**) Transfer and (**b**) output curves of a representative α-Ga_2_O_3_ MOSFET with the OD and HSD contacts. (**c**) Comparison of extracted μ_FE_ for variable L_SD_ = 20, 28, 40 μm from linear (V_DS_ = 1 V) to saturation (V_DS_ = 40 V) region. (**d**) Comparison of extracted R_on_ for variable L_SD_ = 20, 28, 40 μm.

Figure 3a shows transfer characteristics of the representative OD and HSD devices with L_SD_ = 20 μm. The on/off ratios of the devices are 10^6^ at V_DS_ = 10 V. The larger V_DS_ is, the higher the observed off-current is, because the undoped α-Ga_2_O_3_ buffer layer is unintentionally an n-doped layer due to the presence of oxygen vacancies. Therefore, even in the off-state, the source–drain current can flow through the buffer layer (i.e., undoped α-Ga_2_O_3_), which depends on V_DS_. Figure 3b presents the output characteristics of the OD and HSD devices. The R_on_ values of the OD and HSD devices (R_on.OD_, R_on.HSD_) are extracted to be 2.9 and 2.1 kΩ mm, respectively. The varying slopes at low-V_DS_ regions indicate that the current path forms under the Ohmic and then Schottky regions in turn. In low-drain-bias operation, the HSD affects carrier concentration and increases L_GD_ because the Schottky contact is off. The Schottky and Ohmic contact regions are designed to be 34 and 62 µm, respectively. This significant portion of the Schottky contact region results in a longer L_GD_ (i.e., L_SD_ + the width of Schottky contact) under the low-drain-bias regime because the current flows only via the Ohmic region. Therefore, lower drain currents were observed in the low-drain-bias region of output characteristics. Once the Schottky contact region turns on under high drain bias, however, the current drastically increases because of the current flow via the Schottky contact region because the width of the Schottky contact is larger than the transfer length. Thus, a lower R_on_ is extracted in comparison with the conventional Ohmic drain contact. Figure 3c compares extracted field-effect mobility (μ_FE_) of the OD and HSD devices for variable L_SD_ at V_DS_ = 1 V and 40 V. The μ_FE_ is calculated by μ_FE_ = g_m_ · L_SD_/(W · C_OX_ · V_DS_) in the linear region and μ_FE_ = 2 · slope^2^/(C_OX_ · (W/L)) in the saturation region. The oxide capacitance (C_OX_) is calculated to be 712 nF/cm^2^. The μ_FE_ of the HSD device is lower than the OD device at V_DS_ = 1 V because the α-Ga_2_O_3_/Ni Schottky junction was not turned on completely, and this can affect the current’s flow toward the drain [29]. In the saturation region, a high V_DS_ of 40 V induces current paths beneath the Schottky region, and thus similar levels of μ_FE_ were obtained. Figure 3d also compares extracted R_on.OD_ and R_on.HSD_, and R_on.HSD_ is lower than R_on.OD_ regardless of L_SD_.

**Figure 4:**
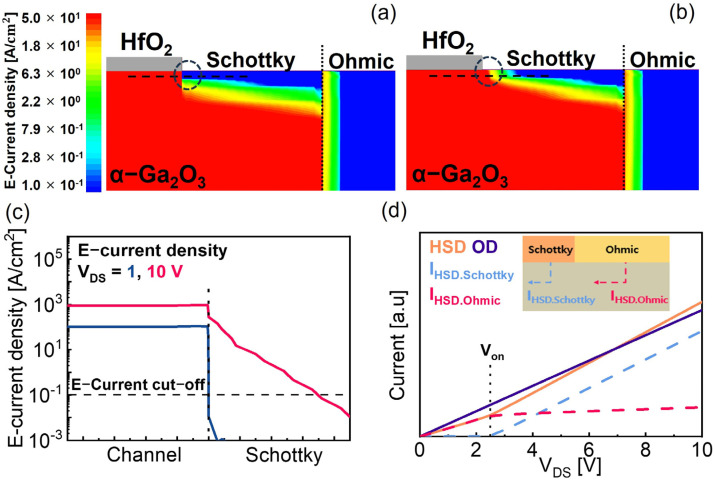
E-current density distribution of the α-Ga_2_O_3_ MOSFET with HSD at (**a**) V_DS_ = 1 V and (**b**) 10 V. Dotted lines and circles are cut−line and edge of the Schottky drain, respectively. (**c**) E-current density line profiles for V_DS_ = 1, 10 V. The E-current cut-off level indicates a minimum value in the color legend. (**d**) Simulated output curves of HSD and OD. Schematic illustrations of two current components through Schottky and Ohmic contact regions are also presented.

Physics-based TCAD simulation was performed to elaborate the turn-on characteristics of the HSD. The simulated OD and HSD devices have same dimensions, including L_SD_ = 40 μm, and the Schottky barrier height of Ga_2_O_3_/Ni was calculated based on reported parameters and was set to 1.2 eV [36,37]. Simulation was conducted taking the bias conditions of V_GS_ = 8 V and V_DS_ = 1 and 10 V. Figure 4a,b depict the E-current density distributions in the vicinity of the HSD contact edge at V_DS_ = 1 and 10 V, respectively. Figure 4a shows that the current flow to the Schottky contact region (I_HSD.Schottky_) is blocked by the depletion of the Schottky region at V_DS_ < V_ON_, and most of the current flows through the Ohmic contact region (I_HSD.Ohmic_). As shown in Figure 4b, however, I_HSD.Schottky_ takes the form of V_DS_ > V_ON_, indicating two parallel current paths. Figure 4c presents E-current density line profiles along the channel, and clearly shows that I_HSD.Schottky_ formed at a V_DS_ of 10 V. The output curves of the simulated HSD and OD devices are presented in Figure 4d. Until a certain point of V_DS_, which is 2.5 V in this simulation, the drain current of the HSD device is mostly composed of I_HSD.Ohmic_. As V_DS_ > 2.5 V, I_HSD.Schottky_ starts to flow and R_on.HSD_ becomes lower than R_on.OD_.

**Figure 5:**
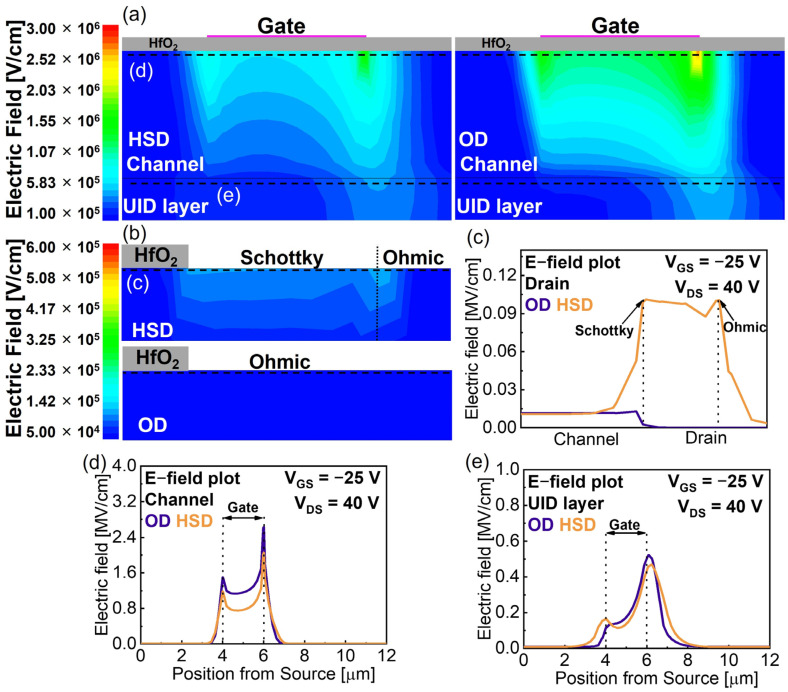
E-field distribution of the α-Ga_2_O_3_ MOSFET with HSD and OD devices near the (**a**) gate and (**b**) drain region. (**c**) Drain E-field line profiles toward lateral axis for OD and HSD. E-field line profiles toward lateral axis for OD and HSD at (**d**) channel and (**e**) UID layer.

To analyze off-state characteristics, additional simulation of the E-field analysis was conducted for both the HSD and OD devices at V_GS_ = −25 V and V_DS_ = 40 V. Figure 5a compares the simulated E-field distribution below the gate electrode of the HSD and OD device. The OD device exhibits a higher peak E-field at the edge of the gate electrode than the HSD device. Figure 5b,c compare the E-field distributions and corresponding line profiles under the drain region, respectively. The HSD device shows a slightly higher peak E-field at the edge of the drain electrode in comparison with the OD device. The difference at the edge of the gate electrode is significant. Figure 5d,e show line profiles of the E-field under the gate electrode and its corresponding α-Ga_2_O_3_/UID interface, respectively. The peak E-field of the HSD device is lower due to the Schottky-contact-induced surface potential change. Additionally, the HSD device exhibits a lower peak E-field at the corresponding region of the α-Ga_2_O_3_/UID interface. Considering the undoped α-Ga_2_O_3_ buffer layer, which is an unintentionally n-doped layer due to the presence of oxygen vacancies, suppressing the peak E-field at the UID layer might contribute to the breakdown characteristics of the α-Ga_2_O_3_ MOSFET [38]. The simulation results foresee enhanced breakdown characteristic from the HSD device, as demonstrated in the following section.

**Figure 6:**
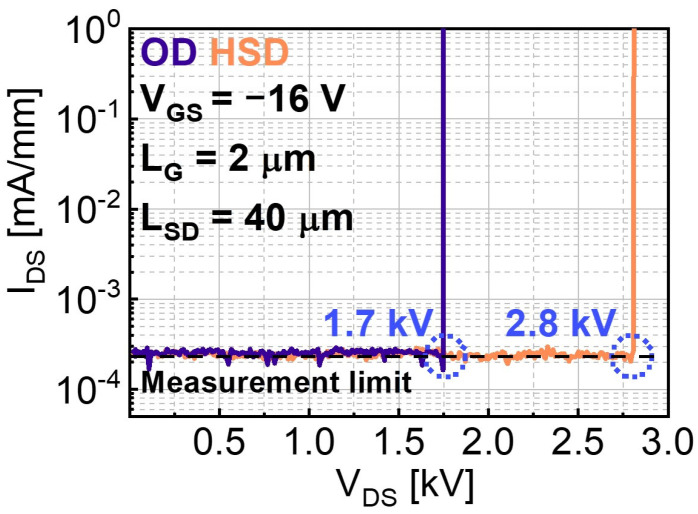
Three terminal off-state breakdown characteristics of α-Ga_2_O_3_ MOSFET with the HSD contact in comparison with the OD device.

Figure 6 shows the three-terminal off-state breakdown measurement results for the OD and HSD devices with L_SD_ = 40 μm (gate-to-drain distance is 34 μm). The devices were submerged in a fluorinert liquid (FC-40) to avoid the effects of air-arcing. V_GS_ was set to −16 V to maintain the off-state. The off-state leakage current level was set by our high-current, high-voltage measurement set-up. V_DS_ was swept until the abrupt increase in I_DS_ was observed, which is indicative of a hard breakdown and defined as BV in this work. The BV of the OD and HSD devices is 1.7 and 2.8 kV, respectively. As discussed above, the Schottky contact region with a smooth interface at the edge of drain electrode functions as a drain field-plate, contributing to electric-field distribution and thus an improved BV [26]. The lateral figures of merit (LFOMs) of the OD and HSD devices are calculated as LFOM = BV^2^/R_on-sp_, where R_on-sp_ is 1.2 and 1 Ω cm^2^, respectively. The LFOMs of the OD and HSD devices are 2.4 and 7.8 MW/cm^2^, respectively. The electrical characteristics of the heteroepitaxial Ga_2_O_3_ MOSFETs are summarized in Table 1 [18,38,39,40,41,42]. Some parameters are missing because they were not discussed in the published results. A record BV of 2.8 kV is achieved for the HSD device, although the R_on-sp_ and LFOM are inferior to those in the report by Jeong et al. [18]. Run-to-run epi growth variations are inevitable on account of the growing conditions of HVPE, and the reproducibility needs to be improved. Still, these results fairly demonstrate that the proposed HSD device can be a promising approach to improve BV as well as R_on-sp_ in comparison with the fabricated OD device. We believe the BV can be further improved by adopting a conventional dielectric passivation layer and a field-plate design.

## 4. Conclusions

In summary, we demonstrated HSD contact for heteroepitaxial α-Ga_2_O_3_ MOSFETs via HVPE on 2-inch (0001) sapphire wafers. The HSD device, comprising both Ti/Al/Ni/Au Ohmic and Ni Schottky electrode regions, exhibits lower R_on_ and similar μ_FE_ for variable channel lengths of 20, 28, and 40 µm in comparison with conventional OD contact. The Schottky and Ohmic regions were modeled as a parallel electrodes, and turn-on characteristics were elaborated through comprehensive TCAD simulation. Furthermore, off-state simulations for the OD and HSD devices were conducted for comparison and analysis of the E-field distribution, as influenced by the Schottky contact region. The HSD device (L_SD_ = 40 μm) exhibits a record BV of 2.8 kV at a R_on-sp_ of 1 Ω-cm^2^, which is superior to the 1.7 kV of the conventional OD device. Our results show that the proposed HSD electrode can be promising with further advanced device structures toward α-Ga_2_O_3_ power devices.

## Figures and Tables

**Table 1:** Comparison of electrical characteristics of heteroepitaxial Ga_2_O_3_ MOSFETs on a sapphire substrate.

Material Growth	Phase	On/Off	μ_FE_ (cm^2^/V-s)	R_on_ (kΩ-mm)	BV (V)
MOCVD [39]	κ-	~10^8^	2.43	--	390
MOCVD [40]	β-	~10^11^	11.4	--	400
MOCVD [38]	β-	~10^5^	150	3.8	240
MOCVD [41]	ε-	--	19	--	--
HVPE [42]	β-	~10^6^	5.3	88.9	155
HVPE [18]	α-	~10^6^	20.4	1.2	2320
HVPE (This work, OD)	α-	~10^6^	20.2	3	1746
HVPE (This work, HSD)	α-	~10^6^	19.2	2.5	2808

## Data Availability

The data presented in this study are available on request from the corresponding author.
